# Supplementary material for: Dynamics and consequences of spliceosome E complex formation
Source: eLife. 2017 Aug 22;6:e27592. doi: 10.7554/eLife.27592 (PMC5779234; doi:10.7554/eLife.27592)
Supplement: Supplementary file 1. [file elife-27592-supp1.docx]

| **RNA #** | **RNA description** | **5**'**SS**^1^ | **BS** | **ΨBS** |
| --- | --- | --- | --- | --- |
| 1 | No functional 5' SS | ca**UA**cc**U** | UACUAAC | UACaAAC |
| 2 | Weak 5' SS | u**GUA**U**G**a | UACUAAC | UACaAAC |
| 3^2^ | Consensus 5' SS | **GGUA**U**GU** | UACUAAC | UACaAAC |
| 4 | Hyperstablized 5' SS mutant | **aGGUAaGUau** | UACUAAC | UACaAAC |
| 5 | No functional 5' SS, BS, and ΨBS mutant | ca**UA**cc**U** | guuagug | guuugug |
| 6 | Weak 5' SS, BS, and ΨBS mutant | u**GUA**u**G**a | guuagug | guuugug |
| 7 | Consensus 5' SS, BS and ΨBS mutant | **GGUA**U**GU** | guuagug | guuugug |
| 8 | Hyperstabilized 5' SS, BS, and ΨBS mutant | **aGGUAaGUau** | guuagug | guuugug |
| 9 | ΨBS mutant | **GGUA**U**GU** | UACUAAC | guuugug |
| 10 | BS mutant | **GGUA**U**GU** | guuagug | UACaAAC |

^1^Nucleotides at positions potentially base pairing to the U1 snRNA are shown in bold.

^2^Full sequence of RNA 3 with the 5SS, BS, ψBS, and ligated biotin handle in bold. The slashes represent the intron boundaries:

5ʹ-GGGAAAGCUAGCUUGGGAUCUCGAGACUAGCAAUAACAAAAUG/**GUAUGU**UAAUAUGGACUAAAGGAGGCUUUUAAGGACACGUAAUAUUGAGUCGACCGUGUUUUUGAUAUCAGUA**UACUAAC**AAGUUGAAUUGCAUU**UACAAAC**UUUUUAUUUUGUAUUGCUUUUCGUCAUUUUAAUAG**/**GGUAGAGUUAGAACCAAGACCGUCAAGCGUGCUUCUAAGGCUUUGAUUGAACGUUACUAUCCAAAGUUGACUUUGGAUUUCCAAACCAACAAGAGACUUUGUGAUGAAAUCGCCACUAUCCAAUCCAAGAGAUUGAGAAACAAGAUUGCUGGUUACACCACCCAUUUGAUGAAGAGAAUCCAAAAGGGUCG**mAmUmCmCmGmGmAmGmCmGmAmG(5-N-U)mAmGmA-Biotin**-3ʹ
