# Supplementary material for: Dynamics and consequences of spliceosome E complex formation
Source: eLife. 2017 Aug 22;6:e27592. doi: 10.7554/eLife.27592 (PMC5779234; doi:10.7554/eLife.27592)
Supplement: Supplementary file 2. [file elife-27592-supp2.docx]

| **Experiment (RNA/WCE)** | **N** | **A(τ_1_)** | **τ_1_ (sec)** | **A(τ_2_)** | **τ_2_ (sec)** | **Corresponding**  **Figure** |
| --- | --- | --- | --- | --- | --- | --- |
| 1/yAAH0055 | 322 | 0.87 ± 0.05 | 14.9 ± 1.4 | 0.13 ± 0.05 | 86.6 ± 27.7 | 1 |
| 2/yAAH0055 | 187 | 0.86 ± 0.04 | 11.3 ± 1.2 | 0.14 ± 0.04 | 141.4 ± 49.8 | 1 |
| 3/yAAH0055 | 449 | 0.46 ± 0.06 | 18.5 ± 3.9 | 0.54 ± 0.06 | 165.0 ± 18.1 | 1, 2, 4 |
| 3/yAAH0055 + Hexokinase | 282 | 0.50 ± 0.08 | 19.4 ± 5.5 | 0.50 ± 0.08 | 175.4 ± 31.7 |  |
| 4/yAAH0055 | 198 | 0.53 ± 0.06 | 16.3 ± 2.9 | 0.47 ± 0.06 | 205.3 ± 28.6 | 1 |
| 7/yAAH0055 + CA | 222 | 0.74 ± 0.07 | 13.4 ± 1.5 | 0.26 ± 0.07 | 64.0 ± 10.0 | 2, 4 |
| 8/yAAH0055 + CA | 179 | 0.34 ± 0.06 | 13.6 ± 2.7 | 0.66 ± 0.06 | 110.1 ± 11.2 | 2 |
| 3/yAAH0055 + CA | 631 | 0.51 ± 0.04 | 20.2 ± 2.7 | 0.49 ± 0.04 | 175.7 ± 14.2 | 2 |
| 7/yAAH0055 | 261 | 0.73 ± 0.05 | 18.0 ± 2.1 | 0.27 ± 0.05 | 148.6 ± 20.4 | 2 |
| 3/yAAH0662 | 211 | 0.61 ± 0.08 | 20.2 ± 3.6 | 0.39 ± 0.08 | 123.7 ± 21.5 | 4, 5 |
| 3/yAAH1317 | 299 | 0.60 ± 0.17 | 52.1 ± 12.4 | 0.40 ± 0.17 | 156.7 ± 35.4 | 4 |
| 7/yAAH1317+ CA | 232 | 0.89 ± 0.10 | 24.5 ± 1.8 | 0.11 ± 0.10 | 167.4 ± 81.8 | 4 |
| 4/yAAH0662 | 156 | 0.57 ± 0.07 | 16.4 ± 4.2 | 0.43 ± 0.07 | 198.9 ± 36.9 | 5 |
| 8/yAAH0662+ CA | 174 | 0.13 ± 0.04 | 16.1 ± 3.7 | 0.87 ± 0.04 | 388.6 ± 46.1 | 5 |
| 3/yAAH0055 + U1 Ablation | 231 | 0.82 ± 0.08 | 14.2 ± 1.5 | 0.18 ± 0.08 | 49.2 ± 9.3 | Fig. 1 Supp. 3 |
| 3/yAAH0055 + Mock Ablation | 215 | 0.44 ± 0.06 | 18.5 ± 4.4 | 0.56 ± 0.06 | 249.6 ± 32.9 | Fig. 1 Supp. 3 |
| 7/yAAH0055 + CA/Mock Ablation | 216 | 0.69 ± 0.06 | 13.2 ± 1.4 | 0.31 ± 0.06 | 80.0 ± 11.9 | Fig. 1 Supp. 3 |
| 3/yAAH0261 | 351 | 0.43 ± 0.06 | 17.4 ± 3.2 | 0.57 ± 0.06 | 147.1 ± 15.3 | Fig. 2 Supp. 3 |
| 7/yAAH0261 | 259 | 0.60 ± 0.07 | 14.1 ± 1.8 | 0.40 ± 0.07 | 66.8 ± 8.6 | Fig. 2 Supp. 3 |
| 8/yAAH0662 | 92 | 0.28 ± 0.05 | 6.2 ± 1.4 | 0.72 ± 0.05 | 439.4 ± 59.3 | Fig. 5 Supp. 2 |
| 4/yAAH0662+ CA | 68 | 0.51 ± 0.07 | 29.4 ± 14.9 | 0.49 ± 0.07 | 365.2 ± 138.9 | Fig. 5 Supp. 2 |
