# Supplementary material for: Dynamics and consequences of spliceosome E complex formation
Source: eLife. 2017 Aug 22;6:e27592. doi: 10.7554/eLife.27592 (PMC5779234; doi:10.7554/eLife.27592)
Supplement: Supplementary file 3. [file elife-27592-supp3.docx]

| **Experiment (RNA/WCE)** | **N** | **A(τ_1_)** | **τ_1_ (sec)** | **A(τ_2_)** | **τ_2_ (sec)** | **Corresponding**  **Figure** |
| --- | --- | --- | --- | --- | --- | --- |
| 3/yAAH01016 | 965 | 0.81 ± 0.02 | 13.5 ±0.7 | 0.19 ± 0.02 | 105.7 ± 11.0 | Fig. 2 Supp. 1 |
| 3/yAAH01016 + Hexokinase | 620 | 0.84 ± 0.04 | 15.8 ± 1.5 | 0.16 ± 0.04 | 126.8 ± 25.7 |  |
| 9/yAAH0106 | 421 | 0.65 ± 0.04 | 13.2 ± 1.2 | 0.35 ± 0.04 | 105.7 ± 13.9 | Fig. 2 Supp. 1 |
| 10/yAAH0106 | 344 | 1 | 9.7 ± 1.9 | NA | NA | Fig. 2 Supp. 1 |
