# Supplementary material for: Dynamics and consequences of spliceosome E complex formation
Source: eLife. 2017 Aug 22;6:e27592. doi: 10.7554/eLife.27592 (PMC5779234; doi:10.7554/eLife.27592)
Supplement: Supplementary file 4. [file elife-27592-supp4.docx]

| **Strain ID** | **Genotype** | **Description** | **Reference** |
| --- | --- | --- | --- |
| yAAH001 | MATa prc1–407 prb1–1122 pep4–3 leu2 trp1 ura3–52 gal2 | BJ2168 | Hoskins *et al*., 2011 |
| yAAH003 | yAAH001 + SNP1::DHFR-HPH + PRP40::DHFR-BLE | Single chromosomal copy of *SNP1* with a 3′ (C-terminal) DHFR tag and hygromycin resistance and a single chromosomal copy of *PRP40* with a 3′ (C-terminal) DHFR tag and nourseothricin resistance | Hoskins *et al*., 2011 |
| yAAH0055 | yAAH001 + SNP1::SNP1-SNAP_f_-HygR+PRP40::PRP40-SNAP_f_-NAT | Single chromosomal copy of *SNP1* with a 3′ (C-terminal) SNAP_f_ tag and hygromycin resistance and a single chromosomal copy of *PRP40* with a 3′ (C-terminal) SNAP_f_ tag and nourseothricin resistance | This work |
| yAAH1153 | yAAH003+ MSL5::MSL5-SNAP_f_-Nat | Double DHFR labeled U1 and SNAP_f_ labeled MSL5 | This work |
| yAAH0450 | yAAH0055 + his3∆::loxP +yhc1::loxP (pAAH0448 URA/CEN)) | Double SNAP_f_ shuffle strain with the chromosomal copy of *YHC1* deleted and a plasmid containing *YHC1* | This work |
| yAAH0662 | yAAH0055 + his3∆::loxP +yhc1::loxP (pAAH0451 TRP/CEN)) | Double SNAP_f_ shuffle strain with the chromosomal copy of *YHC1* deleted and a plasmid containing *YHC1* with an L13F mutation | This work |
| yAAH1317 | yAAH0055 + his3∆::loxP +yhc1::loxP yhc1::loxP (pAAH0628 TRP/CEN)) | Double SNAP_f_ shuffle strain with the chromosomal copy of *YHC1* deletion and a plasmid containing *YHC1* with a D36A mutation | This work |
| yAAH0261 | yAAH0055 + CBP20::URA3 | yAAH0055 with the chromosomal copy of *CBP20* deleted | This work |
| yAAH0106 | yAAH001 + MSL5::MSL5-SNAP_f_-HygR) | Single chromosomal copy of MSL5 the a 3′ (C-terminal) SNAP_f_ tag and hygromycin resistance | This work |
