# Supplementary material for: Dynamics and consequences of spliceosome E complex formation
Source: eLife. 2017 Aug 22;6:e27592. doi: 10.7554/eLife.27592 (PMC5779234; doi:10.7554/eLife.27592)
Supplement: Supplementary file 5. [file elife-27592-supp5.docx]

| **Experiment (RNA/WCE)** | **N** | **A(τ_1_)** | **τ_1_ (sec)** | **A(τ_2_)** | **τ_2_ (sec)** | **Corresponding**  **Figure** |
| --- | --- | --- | --- | --- | --- | --- |
| 3/yAAH1153 | 179 | 0.56 ± 0.07 | 15.9 ± 2.2 | 0.44 ± 0.07 | 118.3 ± 18.2 | Fig. 3 Supp. 5 |
| 9/yAAH1153 | 176 | 0.54 ± 0.08 | 20.7 ± 4.2 | 0.46 ± 0.08 | 165.3 ± 27.9 | Fig. 3 Supp. 5 |
