# Supplementary material for: Dynamics and consequences of spliceosome E complex formation
Source: eLife. 2017 Aug 22;6:e27592. doi: 10.7554/eLife.27592 (PMC5779234; doi:10.7554/eLife.27592)
Supplement: Supplementary file 6. [file elife-27592-supp6.docx]

| **Plasmid ID** | **Plasmid Description** | **Reference** |
| --- | --- | --- |
| pAAH0007 | Contains the DNA template for RNA 1 | Hoskins *et al*., 2011 |
| pAAH0656 | Contains the DNA template for RNA 2 | This work |
| pAAH0016 | Contains the DNA template for RNA 3 | Hoskins *et al*., 2011 |
| pAAH0286 | Contains the DNA template for RNA 4 | This work |
| pAAH0193 | Contains the DNA template for RNA 5 | This work |
| pAAH0615 | Contains the DNA template for RNA 6 | This work |
| pAAH0192 | Contains the DNA template for RNA 7 | This work |
| pAAH0609 | Contains the DNA template for RNA 8 | This work |
| pAAH0448 | pRS316 with WT YHC1 and a URA3 marker | This work |
| pAAH0451 | pRS314 with L13F-YHC1 and a TRP1 marker | This work |
| pAAH0628 | pRS413 with D36A-YHC1 and a HIS3 marker | Schwer *et al*. 2014 |
| pAAH0767 | Contains the DNA template for RNA 9 | This work |
| pAAH0758 | Contains the DNA template for RNA 10 | This work |
